# On Using the BMCSL Equation of State to Renormalize the Onsager Theory Approach to Modeling Hard Prolate Spheroidal Liquid Crystal Mixtures

**DOI:** 10.3390/e23070846

**Published:** 2021-06-30

**Authors:** Donya Ohadi, David S. Corti, Mark J. Uline

**Affiliations:** 1 Department of Chemical Engineering, University of South Carolina, Columbia, SC 29208, USA; donyaohadi@gmail.com; 2 Janssen Research and Development, Welsh and McKean Roads, Spring House, PA 19477, USA; 3 Charles D. Davidson School of Chemical Engineering, Purdue University, West Lafayette, IN 47907, USA; dscorti@purdue.edu; 4 Biomedical Engineering Program, University of South Carolina, Columbia, SC 29208, USA

**Keywords:** liquid crystals, phase transitions, field theory, thermodynamics, entropic control

## Abstract

Modifications to the traditional Onsager theory for modeling isotropic–nematic phase transitions in hard prolate spheroidal systems are presented. Pure component systems are used to identify the need to update the Lee–Parsons resummation term. The Lee–Parsons resummation term uses the Carnahan–Starling equation of state to approximate higher-order virial coefficients beyond the second virial coefficient employed in Onsager’s original theoretical approach. As more exact ways of calculating the excluded volume of two hard prolate spheroids of a given orientation are used, the division of the excluded volume by eight, which is an empirical correction used in the original Lee–Parsons resummation term, must be replaced by six to yield a better match between the theoretical and simulation results. These modifications are also extended to binary mixtures of hard prolate spheroids using the Boublík–Mansoori–Carnahan–Starling–Leland (BMCSL) equation of state.

## 1. Introduction

Systems with hard particle interactions have free energies that are purely entropic. As a result, they provide the simplest non-trivial systems for studying the effects of entropy on, or the entropic control of the thermodynamic and structural phase behavior of various complex fluids. Here, we consider a fluid comprised of hard prolate spheroids that has been used to model lyotropic liquid crystals that exhibit an isotropic–nematic phase transition.

In the 1940s, Lars Onsager originally developed a microscopic theory to capture the isotropic–nematic phase transition of hard cylindrical rods [1]. Onsager’s theory provides an example of a purely entropic phase transition. In contrast to the competition between the energetic and entropic contributions to the free energy that drives the liquid–vapor phase transition, the Onsager theory describes a phase transition that is instead driven by the competition between the orientational and correlational contributions to only entropy [2]. Onsager’s theory has additional historical significance as one of the first examples of a density functional theory of inhomogeneous fluids (in this case, the inhomogeneity arises from the orientations of the particles). Onsager considered suspensions of very long rods that allowed him to express the free energy as a sum of an ideal contribution and an excess term related to the second virial coefficient of two long rods. The second virial coefficient was determined from an integral over the excluded volume or overlap volume of two hard rods. The excluded volume of the two hard rods depends on their relative orientation. By assuming sufficiently large aspect ratios, Onsager was able to obtain a simple analytical expression for the excluded volume of the two long rods in contact as a function of the angle between them [1,2]. The ideal term in the free energy has the lowest numerical value for an isotropic distribution, and the excess term captured by the second viral coefficient decreases as the orientational order increases. The relative weight of the excess term compared to the ideal term increases with increasing density of the fluid until the loss of orientational entropy is compensated by minimization of the rods’ mutual hindrance [2]. The balance between the orientational entropy (favoring the isotropic phase) and the excess term (favoring the nematic phase by lowering the repulsions between the molecules) is determined from functional minimization.

Later work utilizing more sophisticated theoretical techniques [3] and molecular simulations [4] demonstrated that predictions of the Onsager theory are quantitively reliable only for aspect ratios larger than 100. One of the several ways that the Onsager theory has been improved upon is through the determination of more accurate expressions for the excess term, which was accomplished by the inclusion of higher-order viral coefficients, although in an approximate manner [5,6,7]. In particular, the Lee–Parsons resummation method used the Carnahan–Starling (CS) equation of state [8] for hard spheres to effectively include these higher-order virial coefficients [6,7]. The specifics of the Lee–Parsons resummation term are discussed in greater detail in the following section of this manuscript. By including a more accurate excess term to the total free energy of the system, a broader range of systems can be potentially modeled such as rods with smaller aspect ratios and particles with other shapes that are more experimentally relevant, including hard ellipsoids of revolution [9,10,11]. Lee [7] demonstrated that the approximations introduced by the resummation term using the CS equation of state worked extremely well for modeling the isotropic–nematic transition for pure component hard prolate spheroids in comparison to the simulation data. In Reference [7], Lee used the Berne and Pechukas (BP) approximation (also called the Gaussian overlap model) [12] to capture the orientational dependence of the excluded volume of spheroids. 

Despite the success of the Lee–Parsons resummation term and the Gaussian overlap model, recent work has nonetheless focused on generating and evaluating exact expressions for the excluded volume. Such expressions will be presumably necessary when attempting to extend the previously discussed work to mixtures and even more complicated systems (e.g., particles with different shapes). As these exact relations have been analyzed in some detail, though, some interesting inconsistencies have arisen between them and the previous work of Lee. In the sections that follow, we demonstrate that, if a rigorous method is employed to obtain the excluded volume of a given mutual orientation of spheroids [10,11], the division by eight as in the Lee–Parsons resummation term, which is included as an empirical correction and yields excellent agreement when using the Gaussian overlap model approximation, is nonetheless better replaced by a division by six when using the actual (i.e., rigorously obtained) excluded volume. We then demonstrate how to extend the updated resummation term method to the case of mixtures of hard prolate spheroids using the Boublík–Mansoori–Carnahan–Starling–Leland (BMSCL) equation of state [13]. To our knowledge, this is the first time that the BMCSL equation of state has been used for modeling mixtures of liquid crystals. Apart from direct application to prolate spheroids, this analysis should be of importance to several other soft matter systems, including lipid bilayer mechanics [14], liquid–crystalline polymers [3], the modeling of chemical reactions with accurate non-ideal interactions [15], and mixtures of rod-like or ellipsoidal liquid crystal molecules with other shaped particles such as hard spheres [16], to name a few examples.

## 2. Theoretical Methods

### 2.1. Calculating the Excluded Volume of Two Prolate Spheroids Given a Relative Orientation

**Figure 1:**
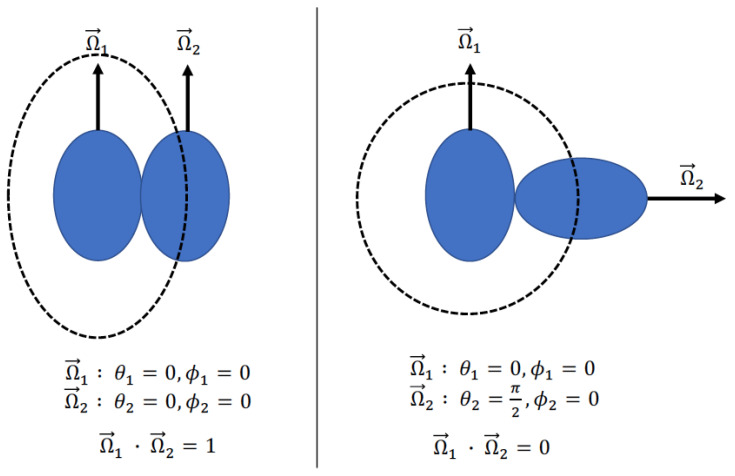
Examples of relative orientations of prolate spheroids and how this maps to the dot product of the spheroids directional unit vectors. The example on the left is for two aligned spheroids. When the spheroid directional unit vectors both point in the same direction, then the dot product is unity, and this relative orientation leads to the lowest excluded volume state. The example on the right is when directional vectors are orthogonal, and this leads to the highest excluded volume state. The dashed lines represent the excluded volume around particle 1 due to particle 2 when they are in a particular relative orientation.

The volume of a single prolate spheroid is $v_{lc}=\frac{4}{3}\pi a^{2}c$, where *a* is the radius of the minor axis and *c* is the radius of the major axis. We define the orientation of a prolate spheroid with a unit vector that points normal to the surface of the spheroid through the major axis. We use two spherical coordinates to define the direction of that unit vector expressed as $\vec{\Omega }=\left(\theta ,\phi \right)$, where θ is the polar angle that is valued from 0 to π and ϕ is the azimuthal angle that runs from 0 to 2π. The differential of $\vec{\Omega }$ is $d\vec{\Omega }=sin\theta d\theta d\phi$. To aid the reader, we provides examples of the relative orientation of two prolate spheroids and how this maps to the dot product of the two unit vectors in Figure 1. In this work, the excluded volume ($v^{ex}$) is the volume around a given particle that is inaccessible to the center of mass of another particle due to the nature of the hard particle interaction potential. When two prolate spheroids of the same size are aligned (${\vec{\Omega }}_{1}\cdot {\vec{\Omega }}_{2}=1$), the excluded volume is $v^{ex}\left({\vec{\Omega }}_{1}\cdot {\vec{\Omega }}_{2}=1\right)=8v_{lc}$. Note that, when two identical perfect spheres are in contact, the excluded volume is also 8 times the volume of a single sphere. In fact, the result that the excluded volume is 8 times the volume of a single spheroid is valid for any two identical spheroidal objects that are aligned (${\vec{\Omega }}_{1}\cdot {\vec{\Omega }}_{2}=1$). This leads to the empirical correction of dividing by 8 that was used in the original resummation term.

As mentioned previously, one widely used method of determining the excluded volume of two prolate spheroids of the same size in any orientation is to use the Gaussian overlap model [12]. The expression for the excluded volume of two spheroids that follows from this model is

(1) $$v^{ex}\left({\vec{\Omega }}_{1}\cdot {\vec{\Omega }}_{2}\right)=8v_{lc}\sqrt{\frac{1-\chi^{2}{\left({\vec{\Omega }}_{1}\cdot {\vec{\Omega }}_{2}\right)}^{2}}{1-\chi^{2}}};\;\mathrm{where}\;\chi =\frac{{\left(c/a\right)}^{2}-1}{{\left(c/a\right)}^{2}+1}.$$

In 1990, Tjipto-Margo and Evans published two papers [10,11] demonstrating how to calculate the exact excluded volume of two ellipsoids of revolution by transforming from center-to-center coordinates to apse vector-based coordinates. We reproduce the required integral for two prolate spheroids here for completeness:

(2) $$v^{ex}\left({\vec{\Omega }}_{1}\cdot {\vec{\Omega }}_{2}\right)=\frac{1}{3}\int_{0}^{2\pi }\int_{0}^{\pi }h_{12}\left\{F\left[F+g_{1}+g_{2}\right]+h_{1}^{\prime \prime }h_{2}^{\prime \prime }\lambda_{ee}^{2}\right\}sin\theta_{k}d\theta_{k}d\phi_{k}\;,$$

where

$$x_{i}=\vec{k}\cdot {\vec{\Omega }}_{i},\;\varepsilon_{i}=\frac{c_{i}^{2}}{a_{i}^{2}}-1\;,\;h_{i}=a_{i}\sqrt{1+\varepsilon_{i}x_{i}^{2}}\;,\;h_{i}^{\prime }=\frac{x_{i}a_{i}^{2}\varepsilon_{i}}{h_{i}}\;,\;h_{i}^{\prime \prime }=\frac{a_{i}^{4}\varepsilon_{i}}{h_{i}^{3}}\;\;,\;\lambda_{ee}=\left({\vec{\Omega }}_{1}\cdot {\vec{\Omega }}_{2}\right)\cdot \vec{k}$$

(3) $$F=h_{1}-x_{1}h_{1}^{\prime }+h_{2}-x_{2}h_{2}^{\prime }\;,\;h_{12}=h_{1}+h_{2}\;,\;g_{i}=\left(1-x_{i}^{2}\right)h_{i}^{\prime \prime }.$$

Equation (2) can be numerically integrated into the desired accuracy.

### 2.2. Onsager Theory with the Lee–Parsons Resummation Term for a Pure Component Liquid Crystal Phase

The residual or excess Helmholtz free energy that follows from the CS equation of state ($F^{CS}$) for a pure component hard sphere fluid is given by [8,17,18]

(4) $$\frac{\beta F^{CS}}{V}=\rho \eta \frac{\left(4-3\eta \right)}{{\left(1-\eta \right)}^{2}},$$

where $\beta =1/k_{B}T$, $k_{B}$ is the Boltzmann constant, T is the absolute temperature, *V* is the volume of the system, ρ is the number density, and $\eta =\rho v_{sphere}$ is the packing fraction. To use Equation (4) to model liquid crystals, Lee redefined the packing fraction to be $\eta =\rho v_{lc}$. Recall that the original Onsager approach [1,2] uses the second virial coefficient to account for the excess free energy. The second virial coefficient for hard particle systems requires an integral of the excluded volume of two hard particles. When the system includes non-spherical molecules, the second virial coefficient requires integrals over the relative orientations of the molecules as well. Thus, to keep the general structure of the excess free energy, Lee [6,7] replaced $v_{lc}$ in $\eta =\rho v_{lc}$ with the average excluded volume of two liquid crystal molecules $\langle v^{ex}\rangle$. Although, considering $\langle v^{ex}\rangle$ > $v_{lc}$, Lee divided by 8 to approximately correct for this additional volume [6,7]. The number 8 has no fundamental significance beyond the fact that, when the hard spheroidal particles are aligned (${\vec{\Omega }}_{1}\cdot {\vec{\Omega }}_{2}=1$), the excluded volume is $v^{ex}\left({\vec{\Omega }}_{1}\cdot {\vec{\Omega }}_{2}=1\right)=8v_{lc}$. However, as recognized by Lee [6], this correction is only true for that one orientation; thus, the fact that it worked so well for the purpose of correcting the resummation term in the free energy is purely coincidental. Finally, for the Lee–Parsons resummation (L-P), Equation (4) becomes:

(5) $$\frac{\beta F^{L-P}}{V}=\frac{1}{2}\rho^{2}\frac{\left(4-3\eta \right)}{z{\left(1-\eta \right)}^{2}}\langle v^{ex}\rangle ,$$

where *z* = 4. We introduce *z* to keep the formal structure of the excess free energy term similar to previous theoretical work on modeling the isotropic–nematic transition. The excess free energy term in the Onsager theory is always presented with a ½ multiplied by the ensemble average excluded volume to maintain the general structure of the second virial coefficient. We factor out the ½ to remain consistent and therefore must introduce *z* in the resummation term. 

Again, by including the resummation term, the higher-order viral coefficients are approximately accounted for in the free energy expression. Hence, with the Lee–Parsons term, the total Helmholtz free energy can be expressed as [6,7,9]

(6) $$\frac{\beta F}{V}=\rho ln\left(\rho \lambda^{3}\right)-\rho +\frac{\rho }{4\pi }\int_{0}^{2\pi }\int_{0}^{\pi }f\left(\theta ,\phi \right)ln\left[f\left(\theta ,\phi \right)\right]sin\theta d\theta d\phi +\;\frac{1}{2}\frac{1}{{\left(4\pi \right)}^{2}}\rho^{2}\frac{\left(4-3\eta \right)}{z{\left(1-\eta \right)}^{2}}\int_{0}^{2\pi }\int_{0}^{\pi }f\left(\theta ,\phi \right)\int_{0}^{2\pi }\int_{0}^{\pi }f\left(\theta^{\prime },\phi^{\prime }\right)v^{ex}\left(\theta ,\phi ,\theta^{\prime },\phi^{\prime }\right)sin\theta^{\prime }d\theta^{\prime }d\phi^{\prime }sin\theta d\theta d\phi +\psi [1-\;\frac{1}{4\pi }\int_{0}^{2\pi }\int_{0}^{\pi }f\left(\theta ,\phi \right)sin\theta d\theta d\phi ],$$

where λ is the de Broglie wavelength, f(θ,ϕ) is a distribution function describing the average orientation of the prolate spheroids, and ψ is a Lagrange multiplier that is used to ensure that the distribution function is properly normalized. The distribution function equals unity for all angles when the liquid crystal system is in the isotropic phase. In addition, the distribution function takes on the appropriate structure when in the nematic phase and when the liquid crystal molecules are aligned along a director.

The equilibrium distribution function for a given packing fraction (or density) is determined by functional minimization. By taking the functional derivative of Equation (6) with respect to f(θ,ϕ) and by setting the result equal to 0, we find that

(7) $$f\left(\theta ,\phi \right)=\frac{exp\left(-\frac{\rho }{4\pi }\frac{\left(4-3\eta \right)}{z{\left(1-\eta \right)}^{2}}\int_{0}^{2\pi }\int_{0}^{\pi }f\left(\theta^{\prime },\phi^{\prime }\right)v^{ex}\left(\theta ,\phi ,\theta^{\prime },\phi^{\prime }\right)sin\theta^{\prime }d\theta^{\prime }d\phi^{\prime }\right)}{q}\;,$$

where

(8) $$q=\frac{1}{4\pi }\int_{0}^{2\pi }\int_{0}^{\pi }exp\left(-\frac{\rho }{4\pi }\frac{\left(4-3\eta \right)}{z{\left(1-\eta \right)}^{2}}\int_{0}^{2\pi }\int_{0}^{\pi }f\left(\theta^{\prime },\phi^{\prime }\right)v^{ex}\left(\theta ,\phi ,\theta^{\prime },\phi^{\prime }\right)sin\theta^{\prime }d\theta^{\prime }d\phi^{\prime }\right)sin\theta d\theta d\phi .$$

The remaining thermodynamic expressions necessary to find the isotropic–nematic phase transition are the chemical potential and pressure. The chemical potential (μ) is determined by taking the derivative of the free energy (Equation (6)) with respect to the density at a constant volume and temperature. The pressure (P) is then determined by subtracting the Helmholtz free energy from the Gibbs free energy. The two expressions are

(9) $$\mu -ln\lambda^{3}=ln\rho -lnq+\frac{1}{2}\frac{1}{{\left(4\pi \right)}^{2}}\eta^{2}\frac{\left(5-3\eta \right)}{v_{lc}z{\left(1-\eta \right)}^{3}}\int_{0}^{2\pi }\int_{0}^{\pi }f\left(\theta ,\phi \right)\int_{0}^{2\pi }\int_{0}^{\pi }f\left(\theta^{\prime },\phi^{\prime }\right)v^{ex}\left(\theta ,\phi ,\theta^{\prime },\phi^{\prime }\right)sin\theta^{\prime }d\theta^{\prime }d\phi^{\prime }sin\theta d\theta d\phi$$

and

(10) $$\beta P=\rho +\frac{1}{2}\frac{1}{{\left(4\pi \right)}^{2}}\left\{\frac{\rho \eta \left(4-3\eta \right)}{v_{lc}z{\left(1-\eta \right)}^{2}}+\frac{\rho \eta^{2}\left(5-3\eta \right)}{v_{lc}z{\left(1-\eta \right)}^{3}}\right\}\int_{0}^{2\pi }\int_{0}^{\pi }f\left(\theta ,\phi \right)\int_{0}^{2\pi }\int_{0}^{\pi }f\left(\theta^{\prime },\phi^{\prime }\right)v^{ex}\left(\theta ,\phi ,\theta^{\prime },\phi^{\prime }\right)sin\theta^{\prime }d\theta^{\prime }d\phi^{\prime }sin\theta d\theta d\phi .$$

The second Legendre polynomial is used to generate the following order parameter (*S*) to assess the ensemble average alignment of uniaxial liquid crystal molecules along a director [2,3,6,7,9]:

(11) $$S=\frac{3}{2}\frac{1}{4\pi }\int_{0}^{2\pi }\int_{0}^{\pi }f\left(\theta ,\phi \right)[cos^{2}\theta ]sin\theta d\theta d\phi -\frac{1}{2}.$$

where *S* is zero in the isotropic phase and non-zero in the nematic phase. A value of unity indicates strict adherence to the orientation of 0 in the polar angle.

### 2.3. Onsager Theory with the Lee–Parsons Resummation Terms Derived from the BMCSL Equation of State for a Liquid Crystal Mixture

Following the same procedure outlined above for introducing the CS equation of state for hard spheres into the Onsager theory for pure hard ellipsoids of revolution, we derive a new resummation procedure that uses the BMCSL equation of state [13,19]. The BMSCL equation of state is formulated similarly to the CS equation of state with a focus on hard sphere mixtures. Our resummation derivation procedure also selects a packing fraction and substitutes an ensemble-averaged excluded volume of two spheroids for the volume of a single spheroid. We also must divide by a constant to correct for the additional volume. The Helmholtz free energy is now written as

(12) $$\frac{\beta F}{V}=\sum_{i=A}^{B}\left\{\rho_{i}ln\left(\rho_{i}\lambda_{i}^{3}\right)-\rho_{i}+\frac{\rho_{i}}{4\pi }\int_{0}^{2\pi }\int_{0}^{\pi }f_{i}\left(\theta ,\phi \right)ln\left[f_{i}\left(\theta ,\phi \right)\right]sin\theta d\theta d\phi \right\}+\;\sum_{i=A}^{B}\sum_{j=A}^{B}\frac{1}{2}\frac{1}{{\left(4\pi \right)}^{2}}\rho_{i}\rho_{j}g_{ij}\int_{0}^{2\pi }\int_{0}^{\pi }f_{i}\left(\theta ,\phi \right)\int_{0}^{2\pi }\int_{0}^{\pi }f_{j}\left(\theta^{\prime },\phi^{\prime }\right)v_{ij}^{ex}\left(\theta ,\phi ,\theta^{\prime },\phi^{\prime }\right)sin\theta^{\prime }d\theta^{\prime }d\phi^{\prime }sin\theta d\theta d\phi +\;\sum_{i=A}^{B}\psi_{i}[1-\frac{1}{4\pi }\int_{0}^{2\pi }\int_{0}^{\pi }f_{i}\left(\theta ,\phi \right)sin\theta d\theta d\phi ],$$

where $g_{ij}$ is the resummation term that follows from the BMCSL equation of state and is given as follows:

$$g_{AA}=\frac{-\ln\left(1-n_{3}\right)}{z\;\eta_{A}}+\frac{3}{z\left(1-n_{3}\right)}+\frac{\eta_{A}}{z}\left[\frac{\ln\left(1-n_{3}\right)}{n_{3}^{2}}+\frac{1}{n_{3}{\left(1-n_{3}\right)}^{2}}\right]$$

$$g_{AB}=\frac{3\frac{\sigma_{A}}{\sigma_{B}}}{z\left(1-n_{3}\right)}+\frac{3\eta_{A}\left(\frac{\sigma_{A}}{\sigma_{B}}\right)}{z}\left[\frac{\ln\left(1-n_{3}\right)}{n_{3}^{2}}+\frac{1}{n_{3}{\left(1-n_{3}\right)}^{2}}\right]$$

$$g_{AB}=\frac{3\frac{\sigma_{A}}{\sigma_{B}}}{z\left(1-n_{3}\right)}+\frac{3\eta_{A}\left(\frac{\sigma_{A}}{\sigma_{B}}\right)}{z}\left[\frac{\ln\left(1-n_{3}\right)}{n_{3}^{2}}+\frac{1}{n_{3}{\left(1-n_{3}\right)}^{2}}\right]$$

(13) $$g_{BB}=\frac{-\ln\left(1-n_{3}\right)}{z\;\eta_{B}}+\frac{3}{z\left(1-n_{3}\right)}+\frac{\eta_{B}}{z}\left[\frac{\ln\left(1-n_{3}\right)}{n_{3}^{2}}+\frac{1}{n_{3}{\left(1-n_{3}\right)}^{2}}\right]\;,$$

where $n_{3}=\eta_{A}+\eta_{B}$ and where $\sigma_{i}=\sqrt[{3}]{8c_{i}a_{i}^{2}}$ is an effective diameter for the prolate spheroid (*i*). In Equation (13), there is a natural asymmetry that occurs from the spheroids having different sizes. 

The equilibrium distribution functions and the thermodynamic variables are derived in the same manner as in the pure component case. The results are

$$f_{A}\left(\theta ,\phi \right)=\frac{exp\left(-\frac{\rho_{A}}{4\pi }g_{AA}\int_{0}^{2\pi }\int_{0}^{\pi }f_{A}\left(\theta^{\prime },\phi^{\prime }\right)v_{AA}^{ex}\left(\theta ,\phi ,\theta^{\prime },\phi^{\prime }\right)sin\theta^{\prime }d\theta^{\prime }d\phi^{\prime }-\frac{1}{2}\left[\frac{\rho_{B}}{4\pi }g_{AB}+\frac{\rho_{B}}{4\pi }g_{BA}\right]\int_{0}^{2\pi }\int_{0}^{\pi }f_{B}\left(\theta^{\prime },\phi^{\prime }\right)v_{AB}^{ex}\left(\theta ,\phi ,\theta^{\prime },\phi^{\prime }\right)sin\theta^{\prime }d\theta^{\prime }d\phi^{\prime }\right)}{q_{A}}$$

$$q_{A}=\frac{1}{4\pi }\int_{0}^{2\pi }\int_{0}^{\pi }exp\left(-\frac{\rho_{A}}{4\pi }g_{AA}\int_{0}^{2\pi }\int_{0}^{\pi }f_{A}\left(\theta^{\prime },\phi^{\prime }\right)v_{AA}^{ex}\left(\theta ,\phi ,\theta^{\prime },\phi^{\prime }\right)sin\theta^{\prime }d\theta^{\prime }d\phi^{\prime }\;-\frac{1}{2}\left[\frac{\rho_{B}}{4\pi }g_{AB}+\frac{\rho_{B}}{4\pi }g_{BA}\right]\int_{0}^{2\pi }\int_{0}^{\pi }f_{B}\left(\theta^{\prime },\phi^{\prime }\right)v_{AB}^{ex}\left(\theta ,\phi ,\theta^{\prime },\phi^{\prime }\right)sin\theta^{\prime }d\theta^{\prime }d\phi^{\prime }\right)sin\theta d\theta d\phi$$

$$\begin{array}{cc} f_{B}\left(\theta ,\phi \right) & \\ & =\frac{exp\left(-\frac{\rho_{B}}{4\pi }g_{BB}\int_{0}^{2\pi }\int_{0}^{\pi }f_{B}\left(\theta^{\prime },\phi^{\prime }\right)v_{BB}^{ex}\left(\theta ,\phi ,\theta^{\prime },\phi^{\prime }\right)sin\theta^{\prime }d\theta^{\prime }d\phi^{\prime }-\frac{1}{2}\left[\frac{\rho_{A}}{4\pi }g_{AB}+\frac{\rho_{A}}{4\pi }g_{BA}\right]\int_{0}^{2\pi }\int_{0}^{\pi }f_{A}\left(\theta^{\prime },\phi^{\prime }\right)v_{AB}^{ex}\left(\theta ,\phi ,\theta^{\prime },\phi^{\prime }\right)sin\theta^{\prime }d\theta^{\prime }d\phi^{\prime }\right)}{q_{B}} \end{array}$$

$$q_{B}=\frac{1}{4\pi }\int_{0}^{2\pi }\int_{0}^{\pi }exp\left(-\frac{\rho_{B}}{4\pi }g_{BB}\int_{0}^{2\pi }\int_{0}^{\pi }f_{B}\left(\theta^{\prime },\phi^{\prime }\right)v_{BB}^{ex}\left(\theta ,\phi ,\theta^{\prime },\phi^{\prime }\right)sin\theta^{\prime }d\theta^{\prime }d\phi^{\prime }\;-\frac{1}{2}\left[\frac{\rho_{A}}{4\pi }g_{AB}+\frac{\rho_{A}}{4\pi }g_{BA}\right]\int_{0}^{2\pi }\int_{0}^{\pi }f_{A}\left(\theta^{\prime },\phi^{\prime }\right)v_{AB}^{ex}\left(\theta ,\phi ,\theta^{\prime },\phi^{\prime }\right)sin\theta^{\prime }d\theta^{\prime }d\phi^{\prime }\right)sin\theta d\theta d\phi$$

$$\begin{array}{cc} \beta \mu_{i}-ln\lambda_{i}^{3}=ln\rho_{i}-lnq_{i}+ & \\ & \sum_{i=A}^{B}\sum_{j=A}^{B}\frac{1}{2}\frac{1}{{\left(4\pi \right)}^{2}}\rho_{i}\rho_{j}{\left(\frac{\partial g_{ij}}{\partial \rho_{i}}\right)}_{\beta ,V,\rho_{k\neq i}}\int_{0}^{2\pi }\int_{0}^{\pi }f_{i}\left(\theta ,\phi \right)\int_{0}^{2\pi }\int_{0}^{\pi }f_{j}\left(\theta^{\prime },\phi^{\prime }\right)v_{ij}^{ex}\left(\theta ,\phi ,\theta^{\prime },\phi^{\prime }\right)sin\theta^{\prime }d\theta^{\prime }d\phi^{\prime }sin\theta d\theta d\phi \end{array}$$

(14) $$\beta P=\rho_{A}+\rho_{B}+\sum_{i=A}^{B}\sum_{j=A}^{B}\frac{1}{2}\frac{1}{{\left(4\pi \right)}^{2}}\left\{\rho_{i}\rho_{j}g_{ij}+\;\rho_{A}\rho_{i}\rho_{j}{\left(\frac{\partial g_{ij}}{\partial \rho_{A}}\right)}_{\beta ,V,\rho_{B}}+\rho_{B}\rho_{i}\rho_{j}{\left(\frac{\partial g_{ij}}{\partial \rho_{B}}\right)}_{\beta ,V,\rho_{A}}\right\}\int_{0}^{2\pi }\int_{0}^{\pi }f_{i}\left(\theta ,\phi \right)\int_{0}^{2\pi }\int_{0}^{\pi }f_{j}\left(\theta^{\prime },\phi^{\prime }\right)v_{ij}^{ex}\left(\theta ,\phi ,\theta^{\prime },\phi^{\prime }\right)sin\theta^{\prime }d\theta^{\prime }d\phi^{\prime }sin\theta d\theta d\phi ,$$

where $v_{i}$ is the volume of molecule *i* and the derivatives are

$$\begin{array}{cc} {\left(\frac{\partial g_{AA}}{\partial \rho_{A}}\right)}_{\beta ,V,\rho_{B}}= & \frac{\ln\left(1-n_{3}\right)v_{A}}{z\eta_{A}^{2}}+\frac{v_{A}}{z\left(1-n_{3}\right)\eta_{A}}+\frac{3v_{A}}{z{\left(1-n_{3}\right)}^{2}}+\frac{v_{A}}{z}\left[\frac{\ln\left(1-n_{3}\right)}{n_{3}^{2}}+\frac{1}{n_{3}{\left(1-n_{3}\right)}^{2}}\right]\\ & +\frac{\eta_{A}v_{A}}{z}\left[-\frac{2\ln\left(1-n_{3}\right)}{n_{3}^{3}}+\frac{2}{n_{3}{\left(1-n_{3}\right)}^{3}}-\frac{1}{n_{3}^{2}\left(1-n_{3}\right)}-\frac{1}{n_{3}^{2}{\left(1-n_{3}\right)}^{2}}\right] \end{array}$$

$$\begin{array}{cc} {\left(\frac{\partial g_{AB}}{\partial \rho_{A}}\right)}_{\beta ,V,\rho_{B}}= & \frac{3\left(\frac{\sigma_{A}}{\sigma_{B}}\right)v_{A}}{z{\left(1-n_{3}\right)}^{2}}+\frac{3\left(\frac{\sigma_{A}}{\sigma_{B}}\right)v_{A}}{z}\left[\frac{\ln n\left(1-n_{3}\right)}{n_{3}^{2}}+\frac{1}{n_{3}{\left(1-n_{3}\right)}^{2}}\right]\\ & +\frac{3\eta_{A}\left(\frac{\sigma_{A}}{\sigma_{B}}\right)v_{A}}{z}\left[-\frac{2\ln\left(1-n_{3}\right)}{n_{3}^{3}}+\frac{2}{n_{3}{\left(1-n_{3}\right)}^{3}}-\frac{1}{n_{3}^{2}\left(1-n_{3}\right)}-\frac{1}{n_{3}^{2}{\left(1-n_{3}\right)}^{2}}\right] \end{array}$$

$${\left(\frac{\partial g_{BA}}{\partial \rho_{A}}\right)}_{\beta ,V,\rho_{B}}=\frac{3\left(\frac{\sigma_{B}}{\sigma_{A}}\right)v_{A}}{z{\left(1-n_{3}\right)}^{2}}+\frac{3\eta_{B}\left(\frac{\sigma_{B}}{\sigma_{A}}\right)v_{A}}{z}\left[-\frac{2\ln\left(1-n_{3}\right)}{n_{3}^{3}}+\frac{2}{n_{3}{\left(1-n_{3}\right)}^{3}}-\frac{1}{n_{3}^{2}\left(1-n_{3}\right)}-\frac{1}{n_{3}^{2}{\left(1-n_{3}\right)}^{2}}\right]$$

$${\left(\frac{\partial g_{BB}}{\partial \rho_{A}}\right)}_{\beta ,V,\rho_{B}}=\frac{v_{A}}{z\left(1-n_{3}\right)\eta_{B}}+\frac{3v_{A}}{z{\left(1-n_{3}\right)}^{2}}+\;\frac{\eta_{B}v_{A}}{z}\left[-\frac{2\ln\left(1-n_{3}\right)}{n_{3}^{3}}+\frac{2}{n_{3}{\left(1-n_{3}\right)}^{3}}-\frac{1}{n_{3}^{2}\left(1-n_{3}\right)}-\frac{1}{n_{3}^{2}{\left(1-n_{3}\right)}^{2}}\right]$$

$${\left(\frac{\partial g_{AA}}{\partial \rho_{B}}\right)}_{\beta ,V,\rho_{A}}=\frac{v_{B}}{z\left(1-n_{3}\right)\eta_{A}}+\frac{3v_{B}}{z{\left(1-n_{3}\right)}^{2}}+\;\frac{\eta_{A}v_{B}}{z}\left[-\frac{2\ln\left(1-n_{3}\right)}{n_{3}^{3}}+\frac{2}{n_{3}{\left(1-n_{3}\right)}^{3}}-\frac{1}{n_{3}^{2}\left(1-n_{3}\right)}-\frac{1}{n_{3}^{2}{\left(1-n_{3}\right)}^{2}}\right]$$

$${\left(\frac{\partial g_{AB}}{\partial \rho_{B}}\right)}_{\beta ,V,\rho_{A}}=\frac{3\left(\frac{\sigma_{A}}{\sigma_{B}}\right)v_{B}}{z{\left(1-n_{3}\right)}^{2}}+\frac{3\eta_{A}\left(\frac{\sigma_{A}}{\sigma_{B}}\right)v_{B}}{z}\left[-\frac{2\ln\left(1-n_{3}\right)}{n_{3}^{3}}+\frac{2}{n_{3}{\left(1-n_{3}\right)}^{3}}-\frac{1}{n_{3}^{2}\left(1-n_{3}\right)}-\frac{1}{n_{3}^{2}{\left(1-n_{3}\right)}^{2}}\right]$$

$$\begin{array}{cc} {\left(\frac{\partial g_{BA}}{\partial \rho_{B}}\right)}_{\beta ,V,\rho_{A}}= & \frac{3\left(\frac{\sigma_{B}}{\sigma_{A}}\right)v_{B}}{z{\left(1-n_{3}\right)}^{2}}+\frac{3\left(\frac{\sigma_{B}}{\sigma_{A}}\right)v_{B}}{z}\left[\frac{\ln\left(1-n_{3}\right)}{n_{3}^{2}}+\frac{1}{n_{3}{\left(1-n_{3}\right)}^{2}}\right]\\ & +\frac{3\eta_{B}\left(\frac{\sigma_{B}}{\sigma_{A}}\right)v_{B}}{z}\left[-\frac{2\ln\left(1-n_{3}\right)}{n_{3}^{3}}+\frac{2}{n_{3}{\left(1-n_{3}\right)}^{3}}-\frac{1}{n_{3}^{2}\left(1-n_{3}\right)}-\frac{1}{n_{3}^{2}{\left(1-n_{3}\right)}^{2}}\right] \end{array}$$

(15) $${\left(\frac{\partial g_{BB}}{\partial \rho_{B}}\right)}_{\beta ,V,\rho_{A}}=\frac{\ln\left(1-n_{3}\right)v_{B}}{z\eta_{B}^{2}}+\frac{v_{B}}{z\left(1-n_{3}\right)\eta_{B}}+\frac{3v_{B}}{z{\left(1-n_{3}\right)}^{2}}+\frac{v_{B}}{z}\left[\frac{\ln\left(1-n_{3}\right)}{n_{3}^{2}}+\frac{1}{n_{3}{\left(1-n_{3}\right)}^{2}}\right]+\frac{\eta_{B}v_{B}}{z}[-\frac{2\ln\left(1-n_{3}\right)}{n_{3}^{3}}+\frac{2}{n_{3}{\left(1-n_{3}\right)}^{3}}-\;\frac{1}{n_{3}^{2}\left(1-n_{3}\right)}-\frac{1}{n_{3}^{2}{\left(1-n_{3}\right)}^{2}}].$$

Finally, the order parameters for each molecule are defined as

(16) $$S_{i}=\frac{3}{2}\frac{1}{4\pi }\int_{0}^{2\pi }\int_{0}^{\pi }f_{i}\left(\theta ,\phi \right)[cos^{2}\theta ]sin\theta d\theta d\phi -\frac{1}{2}.$$

## 3. Results and Discussion

**Figure 2:**
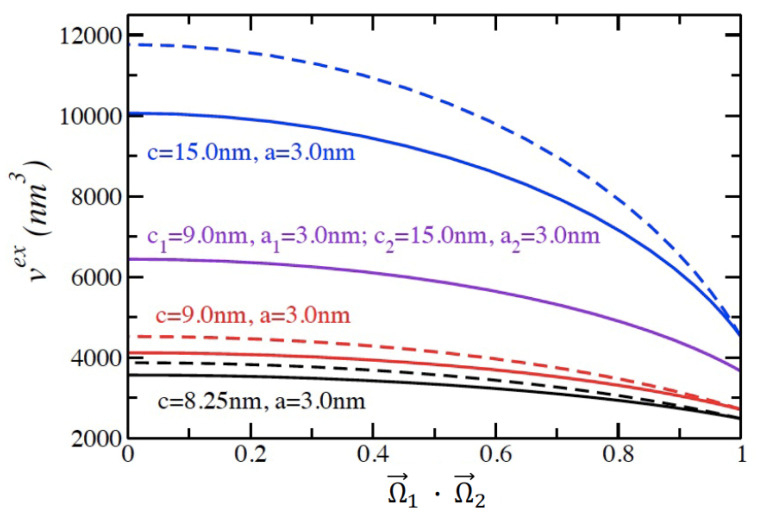
The excluded volume of two prolate spheroids in contact as a function of the dot product of their orientation vectors as determined by the Gaussian overlap method (dashed lines) and a rigorous integration method (solid lines). The purple lines are for a mixture of two prolate spheroids of different sizes.

The excluded volume of two liquid crystal molecules is a fundamental input for the Onsager theory administered with the Lee–Parsons resummation procedure. The excluded volume of two prolate spheroids determined with Equations (1) and (2) are plotted in Figure 2. The approximate Gaussian overlap method (Equation (1)) is plotted with dotted lines, and the rigorous method (Equation (2)) is plotted with solid lines. Note that we are limited to plotting the Gaussian overlap excluded volumes for two prolate spheroids of the same size and that this restriction is not imposed in the rigorous calculation in Equation (2). As discussed in Reference [10], the Gaussian overlap method is consistently higher than the rigorous excluded volume as the dot product between the orientation vectors decreases. The discrepancy increases between the two curves as the aspect ratio increases. The values obtained using Equation (2) were also independently verified using Monte Carlo integration using the contact function developed by Perram and Wertheim [20].

**Figure 3:**
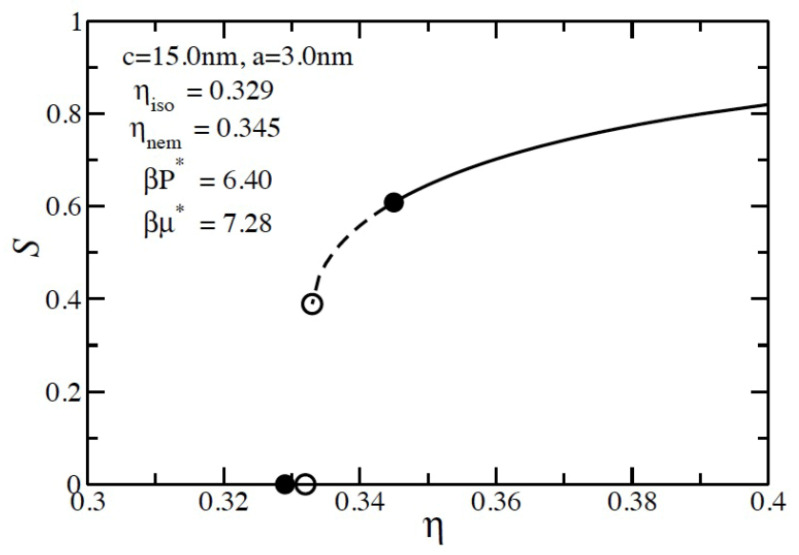
Pure component phase equilibrium for c = 15 nm, a = 3 nm prolate spheroid system. The rigorous excluded volume method was used with z = 3 in the resummation term. *S* is the second Legendre polynomial order parameter described in the text. The dashed curve shows the metastable nematic region. The open symbols mark the limits of metastability, and the closed symbols mark the equilibrium points. The units for pressure and chemical potential are listed in table [1].

A comparison of the two excluded volume calculation methods for pure component liquid crystal systems is shown in Table 1. Two sizes of prolate spheroids (c = 8.25 nm, a = 3.0 nm, and c/a = 2.75; c = 9.0 nm, a = 3.0 nm, and c/a = 3) were chosen to directly for comparison to the simulation data [21]. For our numerical calculation, we used an evenly spaced discretization of 100 values for the polar angle and 50 evenly spaced values for the discretization of the azimuthal angle. When using the Gaussian overlap method, our results agree exactly with those in Reference [7] and agree extremely well with the simulation results when dividing by eight (*z* = 4) in the resummation term. When the rigorous excluded volume is used instead of the Gaussian overlap excluded volume method, the results are no longer in agreement with the simulation results. The lower excluded volume shifts the phase boundary to higher values of the volume fraction, and as a result, there is an increase in the pressure and chemical potential of the phase boundary for both sizes of the prolate spheroid. However, if we divide the resummation term by six (*z* = 3), the rigorous excluded volume method results are in very good agreement with the simulation values. We therefore conclude that, using the correction (*z = 3*), the resummation term should be used whenever accurate values of the excluded volume are used. Figure 3 illustrates the phase equilibrium result for a pure prolate spheroid system with a size of c = 15 nm and a = 3 nm, in which *z =* 3. *S* is the second Legendre polynomial order parameter, as discussed earlier. The solid lines are the equilibrium values, while the dashed lines correspond to the metastable nematic phase. As demonstrated in Table 1, the isotropic–nematic transition occurs at a lower value of the packing fraction due to the larger excluded volume.

**Figure 4:**
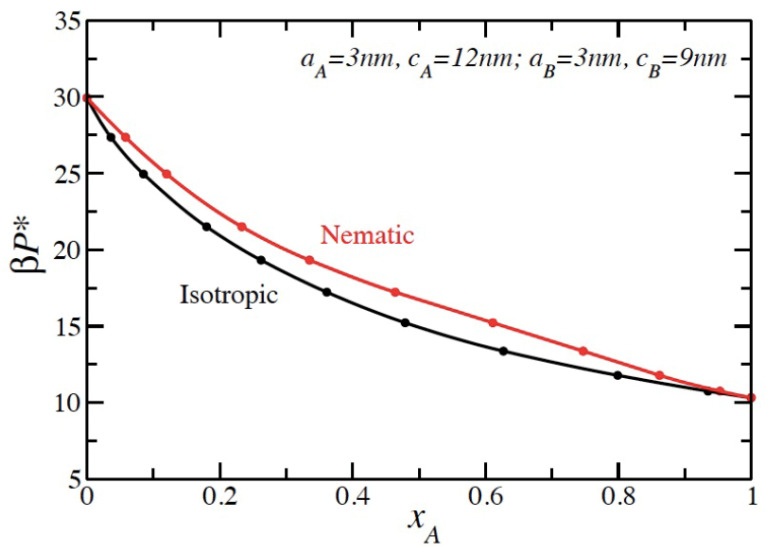
Phase diagram for a mixture of two hard prolate spheroids. Species A is the larger spheroid with c_A_ = 12 nm and a_A_ = 3 nm, and species B has c_B_ = 9 nm and a_B_ = 3 nm. The units for pressure are different from the previous figures and is βP* = βP(8a_A_a_A_c_A_). *x_A_* is the mol fraction of species A.

There are a limited number of prior studies regarding mixtures of hard particle liquid crystal mixtures. As noted previously, there has been some prior work on mixtures of liquid crystal particles mixed with spheres [16], while limited attention has been given to mixtures of hard rods or spherocylinders with different dimensions [22,23,24,25]. These studies found entropy-driven demixing in either the isotropic (leading to an isotropic–isotropic phase transition) or nematic (leading to a nematic–nematic phase transition) phases. In all of these studies, the relative aspect ratios of the molecules needed to be above a certain critical value in order to observe a demixing transition. To use our newly derived BMCSL resummation terms, we chose two prolate spheroids of the same minor axis and moderately different values of the major axis. We selected molecule A with c = 12 nm and a = 3 nm, and molecule B with c = 9 nm and a = 3 nm. The resulting pressure–composition phase diagram is displayed in Figure 4 in which we used the *z* = 3 resummation parameter. The asymmetry in the excluded volume coupled with the asymmetry that is naturally in the BMCSL equation of state yield a phase diagram that favors higher concentrations of molecule A in the nematic phase for the composition space. With the chosen modest difference in the sizes of the two molecules, we did not observe an isotropic or nematic demixing transition.

## 4. Conclusions

The Lee–Parsons resummation term within the Onsager theory was updated for both pure component and mixtures of hard prolate spheroids. If a rigorous method for obtaining the excluded volume of two spheroids is used instead of the approximate Gaussian overlap method, the division by eight (z = 4) in the Lee–Parsons resummation term is better replaced by a division by six (z = 3). We also demonstrated how to extend the updated resummation term method when using the BMSCL equation of state to capture mixtures of hard prolate spheroids. Pure component and mixture phase diagrams for hard prolate spheroids obtained with the updated resummation terms were also presented.

Future work should involve a comprehensive comparison between the new theoretical predictions and new simulation data in order to further validate our theoretical updates. The extension of this work regarding the BMCSL resummation when the relative aspect ratios of the hard prolate spheroid particles are large enough should also be studied in greater detail to determine if nematic and isotropic demixing transitions are indeed observed. In addition, a more robust use of the present work in recent advances in liquid crystal research requires the ability to discretize the excluded volume in layers of varying geometry [9]. This allows the Onsager theory to describe liquid crystal systems in complex spatial geometries [26]. Understanding how to exactly distribute the excluded volume can benefit both simulation and other theoretical methods for studying liquid crystal behavior [27,28].

## Figures and Tables

**Table 1:** Pure component phase equilibrium results for the Lee-Parsons resummation method for two methods of determining the excluded volume. The results for dividing the resummation terms by 8 (z = 4) and by 6 (z = 3) are included for the rigorous excluded volume method. The simulation results are from reference [21]. The units are βP* = βP(8a^2^c), and βμ* = βμ − lnλ^3^.

Pure Component Isotropic-Nematic Transition Data			
Simulation Data [21]	Gaussian Overlap Excluded Volume with z = 4	Rigorous Excluded Volume with z = 4	Rigorous Excluded Volume with z = 3
c = 8.25 nm, a = 3.0 nm	c = 8.25 nm, a = 3.0 nm	c = 8.25 nm, a = 3.0 nm	c = 8.25 nm, a = 3.0 nm
η_iso_ = 0.561 βP* = 30.0	η_iso_ = 0.544 βP* = 25.2	η_iso_ = 0.595 βP* = 38.4	η_iso_ = 0.538 βP* = 29.6
η_nem_ = 0.570 βμ* = 29.96	η_nem_ = 0.552 βμ* = 25.5	η_nem_ = 0.601 βμ* = 37.0	η_nem_ = 0.544 βμ* = 31.9
c = 9.0 nm, a = 3.0 nm	c = 9.0 nm, a = 3.0 nm	c = 9.0 nm, a = 3.0 nm	c = 9.0 nm, a = 3.0 nm
η_iso_ = 0.507 βP* = 18.69	η_iso_ = 0.508 βP* = 19.07	η_iso_ = 0.561 βP* = 28.95	η_iso_ = 0.503 βP* = 22.38
η_nem_ = 0.571 βμ* = 19.27	η_nem_ = 0.517 βμ* = 19.77	η_nem_ = 0.568 βμ* = 28.81	η_nem_ = 0.511 βμ* = 25.01

## Data Availability

The data presented in this study are available through contacting the corresponding author: uline@cec.sc.edu.
